# Medical History from the Earliest Times

**Published:** 1892-11-26

**Authors:** E. T. Withington


### Medical History from the Earliest Times.

By E. T. Withington, M.A., M.B. Oxon.

### XXXIII.—GENERAL SURVEY OF ARABIC MEDICINE.

The extent to which our history has degenerated into anecdote indicates how little there is to tell of actual progress in medicine during this period; but the superstitious awe with which mediæval Europe regarded Arabic science has perhaps been replaced by a somewhat exaggerated depreciation. We are told that the Arabs did nothing but copy and translate from the Greeks, and that those who did this best were not genuine Arabs, but Syrians, Jews, and Persians. In the preceding chapters I have attempted to show that they are not without higher claims, and shall now briefly recapitulate some of their chief services to the healing art.

In an age when no Christian monarch, with the rare exceptions of Charlemagne and Alfred the Great, troubled himself in the slightest about the education of his subjects or the progress of science and literature, we find Caliph after Caliph, and Vizier after Vizier establishing schools and libraries for the public benefit, and filling their courts with physicians and philosophers. Their remarkable generosity to the former would alone entitle them to the gratitude of the profession, and even had the Arabs themselves made no scientific discoveries, they would at least deserve the praise of having encouraged others to do so.

We must here pass over the mysterious Geber, father of chemistry, the *magister magistrorum*, as Friar Bacon called him, as well as the great Moslem botanists, astronomers, and mathematicians, and confine ourselves to the strictly medical sciences. The followers of the prophet looked upon the dissection of human bodies with still greater horror than did the mediæval Christians, and their religious guides held that even the question as to its legality was itself unlawful. Yet it was an Arabic writer who first pointed out the imperfections of the Galenic anatomy. When the physician Abdallatif was in Egypt, the conversation turned one day upon the superiority of observation to mere reading, and someone remarked that there was a great heap of skeletons and dead bodies at Maks. So Abdallatif went there, and found a hill consisting rather of corpses than of earth, and with more than 20,000 skeletons exposed on the surface. The delighted physician proceeded to examine these, and at once noticed that the lower jaw consists of one bone, and not of two as described by Galen. He tells us that he examined 200 lower jaws in every possible way, and got others to examine them also, both in his presence and absence, and they all came to the same conclusion. Similarly he observed that the sacrum is composed of a single bone, and he expresses his intention, "if Providence permits," of writing a book of revised anatomy comparing Galen with nature. No such work, however, has come down to us, and the power of authority in that age cannot be better shown than by the fact that Abdallatif adds, as a sort of postscript, "After all I am not sure that what I have said about the lower jaw is correct."

The Arabs invented the apothecary, whom they called "Sandalani," sandal-wood forming a frequent ingredient both of internal medicines and outward applications, though the peculiar virtues of the oil do not seem to have been recognized. An ambitious or fortunate apothecary sometimes developed into a physician, as is shown by the following anecdote, which also exemplifies the exaggerated importance which the Arabs, and the mediæval physicians after them, paid to uroscopy. Issa al-Sandalani was standing one day at his shop door in Bagdad, when a harem attendant went by carrying a urine glass to a neighbouring physician. "Whose is that?" asked Issa. "Some old woman's." "Say rather the mother of a mighty Prince," replied the joking apothecary. The Caliph al-Mahdi heard the story, and when his favourite wife presented him with a son, the fortunate Issa was astonished to receive £150, two robes of honour, and an appointment at the palace. He was equally prosperous under al-Mahdi's son, Harun al-Rashid. That immortal Caliph, disgusted with the corpulence of his cousin, Issa ben Giafar ben al-Mansur, declared he would give £5,000 to anyone who would make him thin, and that the patient should pay an equal sum. Issa, the apothecary, got the money, but his mode of treatment has unfortunately not been preserved. Arabic physicians seem to have been specially skilful in the treatment of corpulence. Here is another instance: King Sancho, of Leon, was so fat that he could no longer mount a horse, or even walk without support. Wherefore his subjects ridiculed him, and finally deposed and drove him out of the kingdom in the spring of the year 958. He fled, or rather was carried, to his grandmother, Tota, queen of Navarre, who swore to restore him at any cost, even that of alliance with the Saracens; so she sent an embassy to the great Caliph of Cordova, Abdurrahman III., requesting an army and a physician. Both were sent, and both were successful. The Saracen armies were accompanied by apothecaries, for we hear that on one occasion the General Afchin (about A.D. 830) wrote an imaginary prescription, and sent it to all the apothecaries in the camp in order to test their honesty. Some declared they had no such drugs, but others made up the prescription in various ways, and these he dismissed as ignorant pretenders.

Arabia Felix was the land of myrrh and frankincense, and we owe to the Arabs the introduction of several new remedies, of which senna was the chief; but it would be difficult to name half a dozen drugs the use of which certainly originated with them. Some they borrowed from the Hindoos, as, for instance, aconite and mercury, which were employed externally in certain skin diseases, and they were indebted to that people in other sciences as well as medicine. We have seen that the chief Hindoo medical works were early translated into Arabic, and Hindoo physicians were to be found in the ninth century, both at Bagdad and Gondisapor. Harun al-Rashid's uncle, Ibrahim, was once so ill that the Caliph's physician, Gabriel Bachtishua, gave him up, so Giafar, the Vizier, recommended the Hindoo, Sahleh, son of Bahleh. Sahleh declared confidently that he would cure the patient, and the Caliph celebrated this favourable prognosis by a grand banquet. Hardly had he finished when the news came that the prince was dead, and Harun thereupon cursed the grand Vizier, the Hindoo physician, and Hindoos generally, for that he had feasted while his uncle was dying. Then, having drank warm wine, mixed with salt and water, till he got rid of all he had eaten, he went to Ibrahim's house, and sat on the floor mourning. Sahleh, however, declared the prince was still alive, and to prove it ran a pin under his thumb nail, and put something in his nostrils, which made him sneeze. In due time he completely recovered, and Harun confessed that the Hindoos were ever better physicians than the Greeks.

The first pharmacopœia was issued, as we have seen, from the hospital at Gondisapor, but of more importance are the works on materia medica ascribed to Mesue the Younger († 1015), of whom we know little more than the name. These formed the foundation of the western pharmacopœias, were published in more than thirty editions, and were consulted up to the beginning of the last century. In them we find most of the drugs which were either introduced or brought into more general use by the Arabs, such as senna, rhubarb, camphor cloves, cassia, manna, musk, nutmeg, tamarind, cubebs, orange, lemon, gold, pearls, ambergris, bezoar stone, syrups, juleps, and the products of distillation, such as rose-water and alcohol.

Besides the above-mentioned services to medicine, the Arabs also carefully described several new diseases, especially small-pox and measles; we shall see that it was probably from them that the first regulations as to medical education were copied, and above all, that it was their writings which mainly contributed to that brilliant though abortive revival of learning which marks the thirteenth century.

